# Association between estimated glucose disposal rate and major adverse cardiovascular events in patients with type 2 diabetes

**DOI:** 10.1371/journal.pone.0328252

**Published:** 2025-07-17

**Authors:** Maojun Liu, Junyu Pei, Xinqun Hu, Xiaopu Wang

**Affiliations:** 1 Department of Cardiovascular Medicine, The Second Xiangya Hospital, Central South University, Changsha, Hunan, China; 2 The Libin Cardiovascular Institute of Alberta, Cumming school of Medicine, The University of Calgary, Calgary, Alberta, Canada; Tehran University of Medical Sciences, IRAN, ISLAMIC REPUBLIC OF

## Abstract

**Background:**

Estimated glucose disposal rate (eGDR) is associated with risk of adverse outcomes in patients with type 2 diabetes. However, whether eGDR affects long-term glycemic management in patients with type 2 diabetes remains unclear. This study aimed to investigate the relationships between eGDR, long-term visit-to-visit HbA1c variability, and adverse outcomes in patients with type 2 diabetes.

**Methods:**

This post-hoc analysis of the Action to Control Cardiovascular Risk in Diabetes study (ACCORD) included 10,129 type 2 diabetes patients with high cardiovascular risk. Participants were divided into tertiles based on eGDR values. HbA1c variability was assessed using the HbA1c Variability Score (HVS). Primary outcome was major adverse cardiovascular events (MACEs), composite of nonfatal myocardial infarction, nonfatal stroke, and death from cardiovascular causes. Weibull accelerated failure-time models and causal mediation analyses were employed to evaluate relationships between variables.

**Results:**

During a median follow-up of 4.75 years, compared to T1, T3 showed significantly higher risks of MACEs (HR 1.56, 95% CI: 1.27–1.91) and all-cause mortality (HR 1.81, 95% CI: 1.40–2.32) in fully adjusted models. Restricted cubic spline analysis revealed nonlinear relationships between eGDR and outcomes, with risk declining rapidly as eGDR increased until approximately 5 mg/kg/min, after which the curve flattened. A significant negative correlation was observed between HVS and eGDR (β −2.30; 95% CI: −2.65 to −1.95), following a similar nonlinear pattern. In mediation analysis, HVS explained 37.8% of eGDR’s effect on MACEs risk and 21.53% of its effect on all-cause mortality.

**Conclusion:**

In type 2 diabetes patients with high cardiovascular risk, lower eGDR was associated with higher risk of MACEs and all-cause mortality, with HbA1c variability playing an important mediating role in this relationship.

## Introduction

As the global prevalence of hyperglycemia and diabetes continues to increase, it has become increasingly crucial to understand the associations and mechanisms linking these conditions to their most significant complication, cardiovascular disease (CVD) [1–3]. In patients with type 1 and type 2 diabetes, insulin resistance (IR) is reportedly associated with an increased risk of CVD or all-cause mortality [4–6]. Currently, the hyperinsulinemic-euglycemic clamp is recognized as the gold standard for diagnosing IR [7,8]. However, widespread use remains impractical because of its high cost, invasiveness, and limited availability in routine clinical settings. Consequently, numerous indices have emerged that utilize patient clinical characteristics to estimate IR [9–11]. Initially, the estimated Glucose Disposal Rate (eGDR) was employed to assess IR levels in patients with type 1 diabetes, which demonstrated good concordance with the hyperinsulinemic-euglycemic clamp [12–14]. Recent studies have indicated that the eGDR can be used to assess IR levels in patients with type 2 diabetes. Moreover, the results aligned robustly with those of the hyperinsulinemic-euglycemic clamp. Notably, eGDR’s performance significantly surpasses that of the Homeostasis Model Assessment of Insulin Resistance (HOMA-IR) [15].

Recent observational studies on diabetes have demonstrated that higher visit-to-visit HbA1c variability was associated with macrovascular events and microvascular complications [16–20]. However, no studies currently indicate the factors related to higher visit-to-visit HbA1c variability. In this study, we aimed to explore the relationships between eGDR, long-term visit-to-visit HbA1c variability, and adverse outcomes in patients with type 2 diabetes.

## Methods

### Study population

The ACCORD study, which enrolled 10,251 patients with high-risk factors for CVD or with CVD, investigated the effects of intensive blood glucose, blood pressure, and lipid control on cardiovascular risk in patients with type 2 diabetes. The results showed that although strict blood glucose control reduced nonfatal heart events, it was associated with an increased mortality rate. The design and main results of the ACCORD study have been previously published [21–26].

### Ethics approval and consent to participate

Not applicable. We used data from the Action to Control Cardiovascular Risk in Diabetes (ACCORD) Study. ACCORD’s data was obtained from the Biologic Specimen and Data Repository Information Coordinating Center, National Heart, Lung and Blood Institute, U.S. Department of Health & Human Services (URL: https://biolincc.nhlbi.nih.gov/studies/accord/). All data have been de-identified. We describe the data sources as well as a description of data availability in the Methods section of the main text. Thus, no ethical approval was needed for this study.

### Exposure variables and study outcome

We utilized the eGDR as a metric to measure IR, the baseline eGDR was calculated using baseline measurements of waist circumference, HbA1c, and hypertension status obtained at study entry, prior to randomization to intensive or standard glycemic control. The baseline eGDR was calculated using the following formula [15]:


$$eGDR(\frac{mg}{\frac{kg}{\min}})=21.158-\left(0.09*Waist\;circumference\;(cm)\right)-\left(3.407*Hypertension(yes=1/no=0)\right)-\left(0.551*HbA1c\;(\;\%)\right)$$

We divided the population equally into three groups based on the calculated eGDR: T1-Low IR eGDR ≥ 4.35 mg/kg/min, T2 3.09–4.35 mg/kg/min, and T3-High IR eGDR < 3.09 mg/kg/min.

The HbA1c Variability Score (HVS) was used to measure the visit-to-visit variability. HVS was calculated as the percentage of the number of changes in HbA1c > 0·5% (5·5 mmol/mol) compared with the previous follow-up among all HbA1c measurements within an individual [27,28]. HbA1c measurements were collected according to the ACCORD protocol at baseline and approximately every 4 months during follow-up (specifically at months 4, 8, 12, 16, 20, 24, and subsequently every 4 months) [22]. For inclusion in the HVS analysis, participants were required to have a minimum of 3 HbA1c measurements to enable meaningful variability assessment, consistent with previous studies examining HbA1c variability. HVS is much more clinically translatable than the standard deviation (SD) or the coefficient of variation (CV).

The primary outcome was major adverse cardiovascular events (MACEs), a composite of nonfatal myocardial infarction (MI), nonfatal stroke, and death from cardiovascular causes. The secondary outcome was all-cause mortality. The definitions of these events have been published previously [21].

### Statistical analysis

The baseline characteristics of the patients were displayed as frequencies and percentages for categorical variables and as means and standard deviations (SDs) or interquartile ranges (IQRs) for continuous variables, depending on whether the data distribution was normal (assessed using normal Q–Q plots). Categorical variables were compared using χ2 analysis, and continuous variables were analyzed using analysis of variance or the Mann–Whitney U test, according to distribution type.

Three models were constructed to investigate the relationships between IR, HbA1c variability, and outcomes. Model 1 was unadjusted. Model 2 was adjusted for age, sex, ethnicity, CVD history, and treatment arm. Model 3 was the fully adjusted model, adjusted for age, sex, ethnicity, CVD history, treatment arm, body mass index. blood pressure, hyperlipidemia, estimated glomerular filtration rate, comorbidities (heart failure, depression, and albuminuria), and smoking status. We employed the Weibull accelerated failure-time model to estimate the hazard ratios (HRs) for outcomes. We opted not to use the Cox Proportional Hazards model because not all outcomes adhered to the proportional hazard assumption. The Weibull accelerated failure time model presents an alternative strategy for analyzing time-to-event data and may be suitable even when the hazards are not proportional. The Akaike Information Criterion (AIC) and Bayesian Information Criterion (BIC) of the models demonstrate that the Weibull accelerated failure time model fits the data [29]. (Supplemental S1 Table) We utilized a linear regression model to fit the relationship between the eGDR and HVS. Subsequently, we employed restricted cubic splines (RCS) (Model 3) with four knots at the 5th, 35th, 65th, and 95th percentiles to present the nonlinear relationships among the eGDR, HVS, and outcomes. We then used the two-piecewise regression model and bootstrap method to calculate the threshold effect of the relationship between the eGDR, HVS, and outcomes.

**Fig 1:**
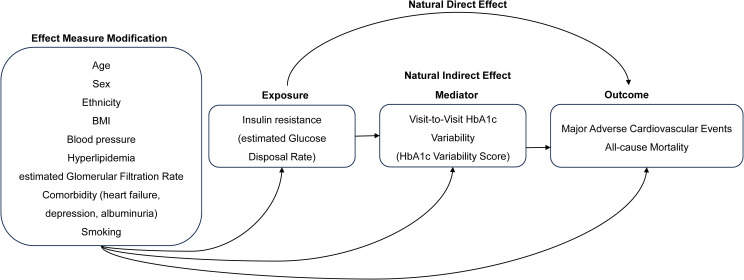
Diagram conceptualizing the relationship of variables in causal mediation analysis.

Mediation analysis is a statistical method used to quantify the impact of exposure on an outcome, as explained by the effect of exposure through the proposed mediating variable. Within a causal mediation framework, the total causal effect (total effect, TE) of exposure on the outcome is decomposed into the natural direct effect (NDE) and natural indirect effect (NIE). Typically, the focus is on the proportional mediation estimate, which denotes the proportion of the TE accounted for by the NDE. This provided a statistical interpretation of the contribution of a specific pathway mediated by the identified variables. We used the extended regression-based causal mediation approach, which allows the inclusion of effect measure modification to conduct a mediation analysis (Model 3, Fig 1) [30–32]. In this study, we hypothesized that visit-to-visit HbA1c variability (measured by the HVS) mediates the impact of IR (measured by the eGDR, with T1 serving as the reference group and T3 serving as the exposure group) on outcomes (Fig 1). Causal mediation estimates were based on the HR scale.

### Sensitivity analysis

Firstly, we conducted the interaction and stratified analyses by age (< 60 and ≥ 60 yrs.), sex, ethnicity, treatment, current smoking status, and CVD history to serve as sensitivity analyses of the relationship between eGDR, HVS, and outcomes. For mediation analysis, we conducted sensitivity analyses by sex, ethnicity, and smoking status, which affected the relationship between eGDR and HVS. Second, we adjusted the time-weighted mean value of HbA1c in our model to explore its effect on the results. All analyses were performed using R Version 4·0·2 (www.R-project.org). *P*-values <0·05 (two-sided) were considered statistically significant.

## Results

### Baseline characteristics

A total of 10,251 participants were included in the ACCORD study; after 122 participants for whom eGDR could not be calculated were excluded, 10,129 were included in this study. Of all the participants in this study (3,901 women), 5,065 were randomly assigned to the intensive glucose treatment group. The mean age was 62.76 years, and the median follow-up was 4.75 years. The incidence rate of MACEs was 2.20 per 100 person-years (95% CI: 2.07–2.34). The study participants’ baseline characteristics stratified by eGDR tertiles are shown in Table 1. Compared with the T1, the T3 had a smaller proportion of women, more Caucasians, and more patients with a history of CVD. However, there was no significant difference in the proportion of patients treated intensively between the three groups, and compared to the T1, the T2 and T3 had a higher percentage of adverse events.

**Table 1:** Baseline characteristics and crude endpoints of the study participants.

eGDR Tertile	Overall participants	T1	T2	T3	*P*-value*
		≥ 4.35 mg/kg/min	3.09-4.35 mg/kg/min	< 3.09 mg/kg/min	
N	10129	3378	3375	3376	
Age	62.76 ± 6.64	63.20 ± 6.90	63.09 ± 6.67	61.97 ± 6.28	<0.01
Female	3901 (38.51%)	1509 (44.67%)	1247 (36.95%)	1145 (33.92%)	<0.01
Race or ethnic group (%)					<0.01
White	6317 (62.37%)	1823 (53.97%)	2156 (63.88%)	2338 (69.25%)	
Black	1917 (18.93%)	619 (18.32%)	676 (20.03%)	622 (18.42%)	
Hispanic	730 (7.21%)	287 (8.50%)	238 (7.05%)	205 (6.07%)	
Other	1165 (11.50%)	649 (19.21%)	305 (9.04%)	211 (6.25%)	
CVD History (%)	3609(35.21%)	1072(31.73%)	1222(36.21%)	1281(37.94%)	<0.01
Education (%)					<0.01
Less than high school	1505 (14.87%)	496 (14.70%)	530 (15.71%)	479 (14.19%)	
High-school graduate	2667 (26.35%)	894 (26.50%)	890 (26.39%)	883 (26.16%)	
Some college	3320 (32.80%)	1025 (30.38%)	1093 (32.40%)	1202 (35.61%)	
College degree or higher	2630 (25.98%)	959 (28.42%)	860 (25.50%)	811 (24.03%)	
Intensive Treatment (%)	5065(50.00%)	1709(50.59%)	1674(49.60%)	1682(49.82%)	0.69
HbA_1c_ measures					
Baseline HbA_1C_ (%)	8.30 ± 1.06	7.89 ± 0.87	8.25 ± 0.94	8.76 ± 1.15	<0.01
HVS (Median, Q1-Q3)	36.36, 21.43-52.94	31.25, 18.18-50.00	35.71, 21.43-52.38	40.00, 25.00-57.14	<0.01
Blood pressure (mm Hg)					
Systolic	136.37 ± 17.11	135.54 ± 16.76	136.85 ± 17.34	136.71 ± 17.20	<0.01
Diastolic	74.88 ± 10.66	73.76 ± 10.31	74.87 ± 10.74	76.03 ± 10.79	<0.01
Heart rate (BPM)	72.65 ± 11.75	71.63 ± 11.17	72.29 ± 12.09	74.04 ± 11.85	<0.01
Body-mass index	32.20 ± 5.41	28.22 ± 4.12	31.82 ± 4.05	36.55 ± 4.44	<0.01
Cholesterol (mg/dl)					
Total	183.26 ± 41.82	183.24 ± 40.78	183.54 ± 41.92	183.01 ± 42.75	0.87
Low-density lipoprotein	104.83 ± 33.86	106.03 ± 33.14	105.02 ± 34.06	103.44 ± 34.32	<0.01
High-density lipoprotein	41.86 ± 11.63	44.07 ± 12.75	41.64 ± 11.20	39.88 ± 10.45	<0.01
Triglyceride (mg/dl)	190.33 ± 148.80	170.99 ± 130.88	191.57 ± 147.27	208.43 ± 164.06	<0.01
Fasting serum glucose (mg/dl)	175.23 ± 56.14	163.64 ± 50.96	172.70 ± 53.92	189.34 ± 60.11	<0.01
Estimated GFR, mL *min^-1^*1.73 m^-2^	91.07 ± 27.18	91.55 ± 29.33	90.11 ± 26.44	91.54 ± 25.62	0.04
Comorbidity (%)					
Protein in urine	2005 (19.80%)	556 (16.46%)	653 (19.35%)	796 (23.59%)	<0.01
Heart failure	490 (4.84%)	119 (3.52%)	148 (4.39%)	223 (6.61%)	<0.01
Neuropathy	2700 (26.66%)	672 (19.89%)	904 (26.79%)	1124 (33.30%)	<0.01
Depression	2396 (23.66%)	634 (18.77%)	791 (23.44%)	971 (28.78%)	<0.01
Eye disease	3155 (31.15%)	965 (28.57%)	1095 (32.44%)	1095 (32.44%)	<0.01
Smoked cigarettes in last 30 days	1415 (13.97%)	534 (15.81%)	429 (12.71%)	452 (13.39%)	<0.01
Crude outcomes (%)					
Primary outcome	1033 (10.20%)	282 (8.35%)	352 (10.43%)	399 (11.82%)	<0.01
All-cause mortality	701 (6.92%)	165 (4.88%)	254 (7.53%)	282 (8.35%)	<0.01
CVD-mortality	327 (3.23%)	67 (1.98%)	125 (3.70%)	135 (4.00%)	<0.01
Non-fatal MI	627 (6.19%)	192 (5.68%)	208 (6.16%)	227 (6.72%)	0.21
Non-fatal Stroke	171 (1.69%)	43 (1.27%)	56 (1.66%)	72 (2.13%)	<0.01
Congestive Heart Failure	438 (4.32%)	87 (2.58%)	139 (4.12%)	212 (6.28%)	<0.01

eGDR, estimated Glucose Disposal Rate. GFR, Glomerular Filtration Rate.

### Association of eGDR with outcomes and visit-to-visit HbA1c variability

**Fig 2:**
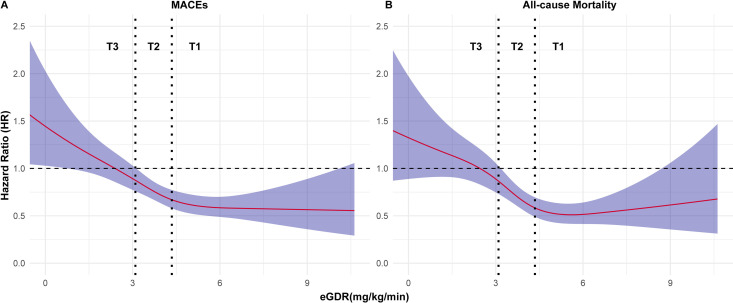
Restricted cubic splines (RCS) revealed the non-linear relationship between eGDR and outcomes. The solid red line is the estimated HR and the purple interval is the 95% confidence interval.

Table 2 presents the relationship between eGDR tertiles and MACEs or all-cause mortality. The risk of MACEs or all-cause mortality increased in the elevated eGDR subgroups, regardless of the model used. T3 has the highest risk of MACEs (hazard ratio [HR] 1.56, 95% CI: 1.27–1.91; model 3, T1 served as reference) and all-cause mortality (HR 1.81, 95% CI: 1.40–2.32; model 3, T1 served as reference). In the RCS analysis, Both MACEs and the risk of all-cause mortality declined rapidly as eGDR rose, and when the eGDR neared 5 mg/kg/min, the curve flattened out afterward (Fig 2, Table 3, Model 3). Two-piecewise regression results showed that the relationship between the eGDR and the risk of MACEs or all-cause mortality was nonlinear (the likelihood ratio test was less than 0.05, Model 3) (Table 3).

**Table 2:** Relationship between eGDR tertiles and outcomes.

eGDR Tertile	Hazard ratio (95% CI) *P*-Value		
	MACEs		
	Model 1^*^	Model 2^#^	Model 3^$^
T1	Ref.	Ref.	Ref.
T2	1.27 (1.09, 1.49) *P* < 0.01	1.19 (1.02, 1.39) *P* = 0.03	1.24 (1.05, 1.47) *P* = 0.01
T3	1.47 (1.26, 1.71) *P* < 0.01	1.41 (1.21, 1.65) *P* < 0.01	1.56 (1.27, 1.91) *P* < 0.01
*P* for trend	<0.01	<0.01	<0.01
	**All-cause mortality**		
T1	Ref.	Ref.	Ref.
T2	1.55 (1.27, 1.88) *P* < 0.01	1.51 (1.24, 1.84) *P* < 0.01	1.47 (1.19, 1.81) *P* < 0.01
T3	1.76 (1.45, 2.13) *P* < 0.01	1.86 (1.53, 2.25) *P* < 0.01	1.81 (1.40, 2.32) *P* < 0.01
*P* for trend	<0.01	<0.01	<0.01

* , model 1 was unadjusted model.

# , model 2, adjusted for age, sex, ethnicity, CVD history and treatment arm.

$ , model 3 was the full-adjusted model, adjusted for age, sex, ethnicity, CVD history, treatment arm, body mass index (BMI), blood pressure, hyperlipidemia, estimated glomerular filtration rate, comorbidity (heart failure, depression, albuminuria), and smoking status.

CI, confidence interval

**Table 3:** Results of the two-piecewise regression model of eGDR and outcomes and HVS.

eGDR (mg/kg/min)	MACEs	All-cause Mortality	HVS
One linear-regression model	0.86 (0.82, 0.91) *P* < 0.01	0.85 (0.80, 0.91) *P* < 0.01	−2.30 (−2.65, −1.95) *P* < 0.01
Inflection point (K, 95%CI)	5.21 (4.71, 5.68)	4.86 (4.60, 5.45)	5.10 (4.87, 5.39)
<K Effect size HR or β (95%CI)	0.82 (0.76, 0.88) *P* < 0.01	0.79 (0.72, 0.86) *P* < 0.01	−3.72 (−4.20, −3.23) *P* < 0.01
>K Effect size HR or β (95%CI)	1.02 (0.90, 1.15) *P* = 0.81	1.03 (0.90, 1.18) *P* = 0.66	0.28 (−0.43, 0.99) *P* = 0.43
Log likelihood ratio test	0.01	<0.01	<0.01

If the p-value of the log-likelihood ratio test is less than 0.05, it supports that the relationship between the dependent and independent variables is curvilinear rather than linear.

HR as well as β is estimated for each 1 unit rise in eGDR.

CI, confidence interval

**Fig 3:**
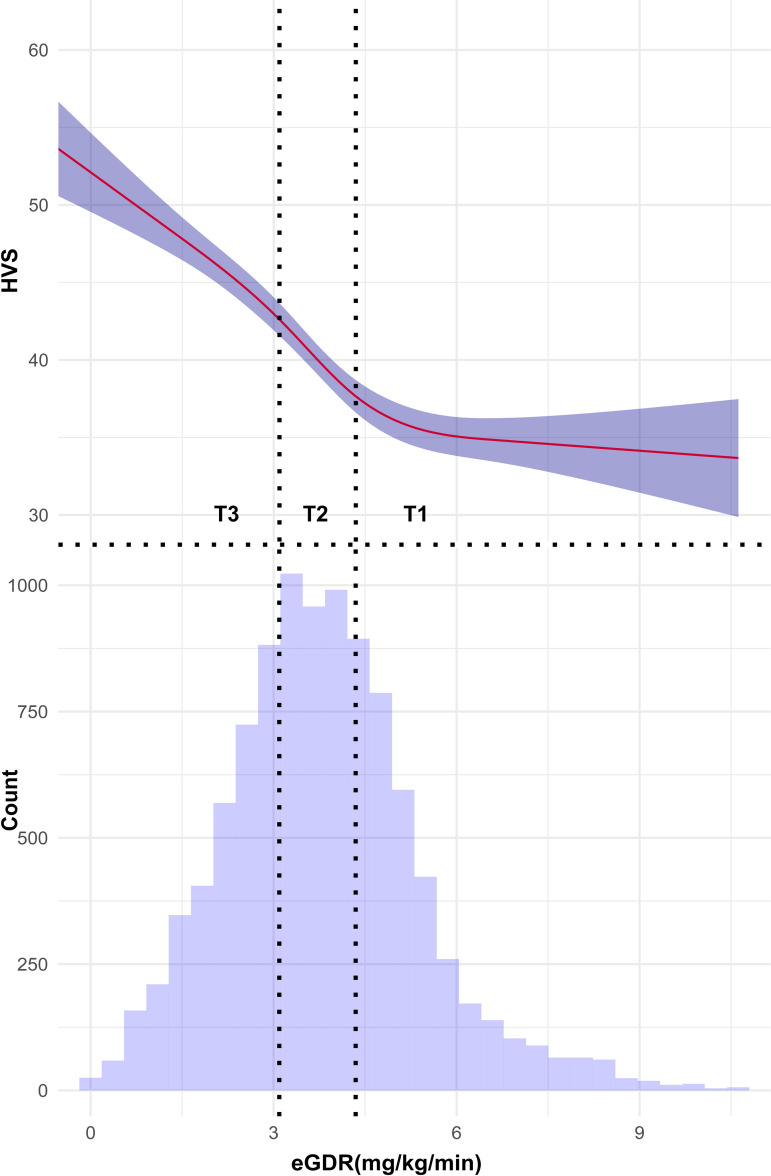
Restricted cubic splines (RCS) revealed the non-linear relationship between eGDR and HVS. Histogram showed the distribution of eGDR. The solid red line is the estimated HR and the purple interval is the 95% confidence interval.

The HVS was significantly different in the eGDR tertiles. Linear regression revealed a significant negative correlation between HVS and eGDR (β −2.30; 95% CI: −2.65 to −1.95; Model 3). Analysis through RCS and two-piecewise regression revealed a comparable trend in the relationship between the eGDR and HVS and that between the eGDR and the risk of MACEs. Specifically, a negative correlation was observed between eGDR and HVS (β −3.72; 95% CI: −4.20 to −3.23; Model 3) when eGDR was below the inflection point (5.10, 95% CI: 4.87–5.39, Loglikelihood ratio test<0.01, Model 3). However, beyond this inflection point, an increase in the eGDR did not reduce the HVS (Fig 3, Table 3).

An elevated HVS was significantly associated with an elevated risk of MACEs or all-cause mortality. The highest HVS subgroup (HVS ≥ 80) had a 1.58-fold risk of MACEs (HR 1.58, 95% CI: 1.04–2.42; Model 3) and a 3.71-fold risk of all-cause mortality (HR 3.71, 95% CI: 2.41–5.71; Model 3) relative to reference (HVS < 20) (Supplemental S2 Table). The trend between the HVS and the risk of MACEs or all-cause mortality was a J-shaped curve (Supplemental S3 Fig.).

### Causal mediation analyses

In the causal mediation analysis (Model 3), we used T1 as the reference group and T3 as the exposure group. We fit a linear mediator model and a Weibull regression outcome model. Table 2 presents the causal mediation results.

For the MACEs, in the overall cohort HVS mediator analysis, the TE HR estimate was 1.38 (95% CI: 1.13–1.69). The NDE HR estimate was 1.21 (95% CI: 0.97–1.50), and the NDE was not statistically significant, whereas the NIE HR (mediated) estimate was 1.14 (95% CI: 1.10–1.19). The proportion of mediated estimates was 37.8%. This indicated that the direct effect of eGDR on the risk of MACEs was not statistically significant in the overall population (P = 0.09); however, the effect on the risk of MACEs through HVS was statistically significant (P < 0.01), where HVS explained 37.8% of the effect of eGDR on the risk of MACEs (P = 0.02). In the case of intensive blood glucose treatment, the results of the causal mediator analysis were similar to those in the overall population. However, the direct effect of eGDR on the risk of MACEs was significant in the context of standard glucose treatment (HR, 1.23; 95% CI: 1.01–1.51), unlike intensive glucose treatment. (Table 4)

**Table 4:** Causal mediation analysis estimates* decomposing the total effect HR of eGDR T3 on MACEs and all-cause mortality compared with T1 into the natural direct (non-mediated) effect and natural indirect (mediated) effect by HVS.

eGDR T3 *vs.* T1	Hazard ratio (95% CI) *P*-Value	
	MACEs	All-cause mortality
	Mediator: HVS	Mediator: HVS
Overall		
Total effect	1.38 (1.13, 1.69) *P* < 0.01	1.51 (1.26, 1.81) *P* < 0.01
Natural direct effect	1.21 (0.97, 1.50) *P* = 0.09	1.36 (1.13, 1.65) *P* < 0.01
Natural indirect effect	1.14 (1.10, 1.19) *P* < 0.01	1.11 (1.08, 1.15) *P* < 0.01
% mediated	37.80, *P* = 0.02	21.53, *P* < 0.01
Standard blood glucose management		
Total effect	1.41 (1.17, 1.71) *P* < 0.01	1.47 (1.25, 1.74) *P* < 0.01
Natural direct effect	1.23 (1.01, 1.51) *P* = 0.04	1.31 (1.10, 1.56) *P* < 0.01
Natural indirect effect	1.15 (1.10, 1.19) *P* < 0.01	1.12 (1.09, 1.16) *P* < 0.01
% mediated	35.44, *P* = 0.01	25.68, *P* < 0.01
Intensive blood glucose management		
Total effect	1.35 (1.08, 1.69) *P* < 0.01	1.53 (1.26, 1.86) *P* < 0.01
Natural direct effect	1.18 (0.93, 1.49) *P* = 0.17	1.36 (1.11, 1.67) *P* < 0.01
Natural indirect effect	1.14 (1.10, 1.19) *P* < 0.01	1.12 (1.09, 1.16) *P* < 0.01
% mediated	41.50, *P* = 0.04	22.89, *P* < 0.01

The total effect hazard ratio (HR) represents the overall effect of T3 compared with T1 on the adverse outcomes. It decomposes as follows: (total effect HR) = (natural direct effect HR) × (natural indirect effect HR). CI, confidence interval.

* , Conditioning on overall population median age, body mass index, blood pressure, lipid profile, Estimated Glomerular Filtration Rate, and White, male, non-smoker, free of comorbidities.

For all-cause mortality risk, in the overall cohort HVS mediator analysis, the TE estimate was 1.51 (95% CI: 1.26–1.81), the NDE estimate was 1.36 (95% CI: 1.13–1.65), and the NIE estimate was 1.11 (95% CI: 1.08–1.15). Unlike the risk of MACEs, the effect of the eGDR on the risk of all-cause mortality was significant for both NDE (P < 0.01) and NIE (P < 0.01). The proportion of the mediated estimate was 21.53% (P < 0.01). The results between the two groups, classified according to whether intensive blood glucose treatment was used, were similar to those of the overall participants (Table 4).

### Sensitivity analysis

Supplemental S4 Fig shows the results of the interaction and stratified analyses. Sex, race, and smoking status influenced the relationship between the eGDR and HVS (P_Interaction_<0.05). However, no factor played an interactive role in the association between the eGDR and MACEs. Moreover, the main results did not change significantly after adjusting for the time-weighted mean HbA1c in our model, and subgroup analysis stratified by time-weighted mean HbA1c levels (≤7% vs > 7%) showed consistent associations, indicating that our results remain robust regardless of time-weighted mean HbA1c. (Supplemental S5–S7 Table).

Regardless of the subgroup, eGDR was negatively associated with the risk of HVS or MACEs. We then adjusted the parameters of the causal mediation analysis according to sex, ethnicity, and smoking status and found that these factors did not affect the results of the causal mediation analysis (Supplemental S8–S11 Table).

## Discussion

Our *post-hoc* analysis of data from the ACCORD study participants with type 2 diabetes at elevated cardiovascular risk showed that eGDR was strongly associated with the risk of MACEs or all-cause mortality and that visit-to-visit HbA1c variability assessed by the HVS significantly mediated this association. To the best of our knowledge, this is the first study to demonstrate that eGDR is associated with visit-to-visit HbA1c variability and that visit-to-visit HbA1c variability mediated the association of eGDR and adverse outcomes.

IR is the inability of insulin to exert its normal effects in insulin-sensitive target tissues such as skeletal muscle, adipose tissue, liver, and pancreas. These tissues are the primary targets of insulin for glucose metabolism [5]. IR can lead to alterations in glucose metabolism, chronic hyperglycemia, and abnormalities in lipid metabolism, resulting in chronic inflammation, oxidative stress, and endothelial dysfunction, potentially leading to cellular damage and atherosclerosis [33]. Furthermore, an inverse relationship between IR and insulin secretion has been confirmed. Therefore, an increase in secretion from β-cells can compensate for decreased insulin sensitivity. As a result, insulin secretion increases in direct proportion to IR to maintain the glucose metabolism balance [34,35]. In patients with type 2 diabetes, IR does not worsen with the onset of hyperglycemia; however, insulin secretion decreases over time. Moreover, insulin may be associated with an elevated CVD risk in patients with type 2 diabetes [36]. In summary, IR may be one of the reasons why improved glycemic control did not reduce the risk of macrovascular complications in patients with type 2 diabetes. However, the challenges IR poses in blood glucose management are unclear.

None of the studies (such as the ACCORD study [24], the Action in Diabetes and Vascular Disease: Preterax and Diamicron MR Controlled Evaluation (ADVANCE) study [37], and the Veterans Administration Diabetes Trial (VADT) [38]) designed to examine intensive glucose management have yielded positive results. Consequently, this prompted the following question: In addition to hyperglycemia, what other factors contribute to elevated cardiovascular risk in patients with type 2 diabetes? Notably, an analysis based on VADT suggested that in the intensive glucose control group, higher HbA1c variability was associated with an increased risk of cardiovascular events, whereas this was not the case in the standard control group [39]. This study indicated that increased HbA1c variability may offset the cardiovascular benefits of a sustained 1.5% (16.4 mmol/mol) reduction in HbA1c levels during the study period [38]. This evidence suggests that visit-to-visit HbA1c variability plays an important role in the long-term risk management of patients with type 2 diabetes. Many previous studies have shown that patients with higher visit-to-visit HbA1c variability have more risk factors at baseline [27,28]. The association between the risk of adverse events and HbA1c variability may better reflect baseline differences in patient characteristics than a direct attribute of HbA1c variability. However, no relevant studies have clarified the factors associated with visit-to-visit HbA1c variability.

Consistent with previous studies [15,28], both an elevated HVS and reduced eGDR were associated with an elevated risk of MACEs or all-cause mortality, even after adjusting for the time-weighted HbA1c average. We also found a negative correlation between eGDR and HVS. Their relationship was not linear; when eGDR was less than 5.1 mg/kg/min, HVS decreased as eGDR increased, and when eGDR was greater than 5.1 mg/kg/min, eGDR had no significant effect on HVS. The shape of the fitted curve between the eGDR and HVS was very similar to that between the eGDR and MACEs risk. No relevant studies have demonstrated the relationship between IR and visit-to-visit HbA1c variability. We believe that one possible reason why IR leads to higher visit-to-visit HbA1c variability is that, in the ACCORD study, patients’ blood glucose was mainly controlled with sulfonylureas and insulin, and as the degree of IR deepened, the effect of sulfonylureas and insulin on glycemic control gradually diminished; thus, the general population showed a tendency for HVS to increase as the eGDR decreased. Although our study was the first to suggest that IR may contribute to elevated HbA1c variability, the emergence of novel hypoglycemic agents in the last decade (especially Sodium-Glucose Co-Transporter 2 inhibitors (SGLT2i) and Glucagon-Like Peptide-1 Receptor Agonists (GLP-1RA), whose mechanism of lowering blood glucose is different from that of sulfonylureas) have led to the fact that the current management of patients with type 2 diabetes is different from that used in the ACCORD study; although second-generation sulfonylureas or insulin are still widely used for glycemic management in patients with type 2 diabetes, more research is needed in patients with newer hypoglycemic agents (such as SGLT2i and GLP-1RA).

In our study, we found that the HVS significantly mediated the association of the eGDR and the risk of adverse outcomes in both the intensive and standard treatment groups. Interestingly, the effect of the NDE of eGDR on the risk of MACEs was not significant in the overall population or the intensive treatment group; however, the effect of NIE via HVS was statistically significant. This may be because the glucose-lowering mechanisms of the sulfonylureas and insulin-based glucose-lowering agents used in the ACCORD study were associated with an interaction between eGDR, as noted above, and that intensive treatment may have strengthened this association of IR increasing the risk of MACEs by increasing HbA1c variability. Our results remained robust after adjusting for the time-weighted HbA1c average. However, for the risk of all-cause mortality, both NDE for eGDR and NIE via HVS were significant in both the intensive and standard treatment groups. These results suggest that the variability of HbA1c in patients with type 2 diabetes plays an important role in the relationship between eGDR and the risk of adverse outcomes. Attempts to use more intensive monitoring of the glycemic profile in patients with more severe IR may be able to reduce the risk of adverse outcomes in this group of patients. However, because of the approach to glycemic management used in the ACCORD study, further research is needed to determine whether the results of our study can be applied to patients treated with newer hypoglycemic agents.

Our study had several advantages. First, we used the latest regression-based causal mediation analysis, which allowed us to incorporate effect measure modifications when conducting the causal mediation analysis [31]. Second, the sample size was large, providing adequate statistical power for our analyses. However, this study has some important limitations that warrant careful consideration. First, because our study was a post-hoc analysis of the ACCORD trial, it has inherent limitations typical of secondary analyses. The ACCORD study was not originally designed to investigate the relationship between insulin resistance and cardiovascular outcomes in patients with type 2 diabetes, which limits our ability to establish definitive causal relationships. While our mediation analysis provides insights into potential pathways, the observational nature of these associations requires cautious interpretation, and prospective studies specifically designed to test these hypotheses are needed for validation. Second, as mentioned above, the glucose-lowering strategies used in the ACCORD study (primarily sulfonylureas and insulin) differed from current strategies for managing blood glucose in patients with type 2 diabetes, which increasingly utilize SGLT2 inhibitors and GLP-1 receptor agonists. However, considering that insulin and sulfonylureas are still widely used for glycemic management in patients with type 2 diabetes globally, our results retain clinical relevance, although validation studies are needed in patients treated with newer hypoglycemic agents. Finally, due to the non-linear relationship between eGDR and HVS, current causal mediation effect models cannot adequately fit such complex non-linear relationships. Therefore, we utilized categorized eGDR values to approximate a more linear relationship between these variables, which may not fully capture the complexity of these associations.

## Conclusion

In patients with type 2 diabetes at high cardiovascular risk, lower eGDR was associated with higher risk of MACEs and all-cause mortality, with HbA1c variability playing an important mediating role in this relationship. However, given the post-hoc nature of this analysis, these findings should be interpreted as associations rather than definitive causal relationships, and prospective validation studies are warranted to confirm these observations and their clinical implications.

## Supporting information

S1 Table Akaike Information Criterion and Bayesian Information Criterion of different models. (DOCX)

S2 Table Relationship between HVS group and outcomes. (DOCX)

S3 Fig Restricted cubic splines (RCS) revealed the non-linear relationship between HVS and outcomes. The solid red line is the estimated HR and the purple interval is the 95% confidence interval.
(DOCX)

S4 Fig β or hazard ratios per 1 standard deviation–increase in eGDR for HVS and major adverse cardiovascular events. Each stratification was adjusted for all factors in model 3 except for the stratification factor itself. CI, confidence interval.
(DOCX)

S5 Table Relationship between HVS group and outcomes stratifying participants by time-weighted mean value of HbA1c (≤7% vs > 7%) during the trial period. (DOCX)

S6 Table Causal mediation analysis estimates* after including time-weighted mean value of HbA1c. (DOCX)

S7 Table Relationship between eGDR tertiles, HVS group and outcomes after adjusting time-weighted mean value of HbA1c. (DOCX)

S8 Table Causal mediation analysis estimates*, by conditioning on female. (DOCX)

S9 Table Causal mediation analysis estimates*, by conditioning on Black people. (DOCX)

S10 Table Causal mediation analysis estimates*, by conditioning on ethnicity (non-White and non-Black). (DOCX)

S11 Table Causal mediation analysis estimates*, by conditioning on smoker. (DOCX)
