# Supplementary material for: Association between estimated glucose disposal rate and major adverse cardiovascular events in patients with type 2 diabetes
Source: PLoS One. 2025 Jul 17;20(7):e0328252. doi: 10.1371/journal.pone.0328252 (PMC12270132; doi:10.1371/journal.pone.0328252)
Supplement: S1 Table — (DOCX) [file pone.0328252.s001.docx]

**S1 Table. Akaike Information Criterion and Bayesian Information Criterion of different models.**

| Outcome |  | AIC | BIC |
| --- | --- | --- | --- |
| MACES | Cox regression model | 17070.05 | 17199.55 |
|  | Weibull regression model | 7690.432 | 7834.319 |
| All-cause mortality | Cox regression model | 10843.38 | 10972.88 |
|  | Weibull regression model | 4837.376 | 4981.262 |

AIC, Akaike Information Criterion. BIC, Bayesian Information Criterion.
