# Supplementary material for: Association between estimated glucose disposal rate and major adverse cardiovascular events in patients with type 2 diabetes
Source: PLoS One. 2025 Jul 17;20(7):e0328252. doi: 10.1371/journal.pone.0328252 (PMC12270132; doi:10.1371/journal.pone.0328252)
Supplement: S2 Table — (DOCX) [file pone.0328252.s002.docx]

**S2 Table. Relationship between HVS group and outcomes.**

| HVS Group | Hazard ratio (95% CI) *P*-Value | | |
| --- | --- | --- | --- |
|  | **MACEs** | | |
|  | Model 1* | Model 2# | Model 3$ |
| 0-20 | Ref. | Ref. | Ref. |
| 20-40 | 1.06 (0.87, 1.29) *P=*0.56 | 1.07 (0.87, 1.30) *P*=0.52 | 0.99 (0.81, 1.22) *P*=0.96 |
| 40-60 | 1.84 (1.52, 2.22) *P*<0.01 | 1.91 (1.57, 2.33) *P*<0.01 | 1.76 (1.44, 2.15) *P*<0.01 |
| 60-80 | 1.90 (1.53, 2.37) *P*<0.01 | 2.10 (1.68, 2.65) *P*<0.01 | 1.94 (1.54, 2.45) *P*<0.01 |
| 80-100 | 1.63 (1.08, 2.46) *P*=0.02 | 1.79 (1.18, 2.72) *P*<0.01 | 1.58 (1.04, 2.42) *P*=0.03 |
| *P* for trend | <0.01 | <0.01 | <0.01 |
|  | **All-cause Mortality** | | |
| 0-20 | Ref. | Ref. | Ref. |
| 20-40 | 1.00 (0.78, 1.28) *P=*0.99 | 1.08 (0.84, 1.39) *P=*0.53 | 1.00 (0.79, 1.28) *P=*1.00 |
| 40-60 | 1.50 (1.18, 1.90) *P*<0.01 | 1.84 (1.44, 2.36) *P*<0.01 | 1.69 (1.32, 2.17) *P*<0.01 |
| 60-80 | 1.87 (1.42, 2.45) *P*<0.01 | 2.58 (1.94, 3.43) *P*<0.01 | 2.34 (1.75, 3.11) *P*<0.01 |
| 80-100 | 2.77 (1.84, 4.19) *P*<0.01 | 4.15 (2.72, 6.33) *P*<0.01 | 3.71 (2.41, 5.71) *P*<0.01 |
| *P* for trend | <0.01 | <0.01 | <0.01 |

*, model 1 was unadjusted model.

#, model 2, adjusted for age, sex, ethnicity, CVD history and treatment arm.

$, model 3 was the full-adjusted model, adjusted for age, sex, ethnicity, CVD history, treatment arm, body mass index (BMI), blood pressure, hyperlipidemia, estimated glomerular filtration rate, comorbidity (heart failure, depression, albuminuria), and smoking status.

CI, confidence interval.
