# Supplementary material for: Association between estimated glucose disposal rate and major adverse cardiovascular events in patients with type 2 diabetes
Source: PLoS One. 2025 Jul 17;20(7):e0328252. doi: 10.1371/journal.pone.0328252 (PMC12270132; doi:10.1371/journal.pone.0328252)
Supplement: S3 Fig — The solid red line is the estimated HR and the purple interval is the 95% confidence interval. (DOCX) [file pone.0328252.s003.docx]

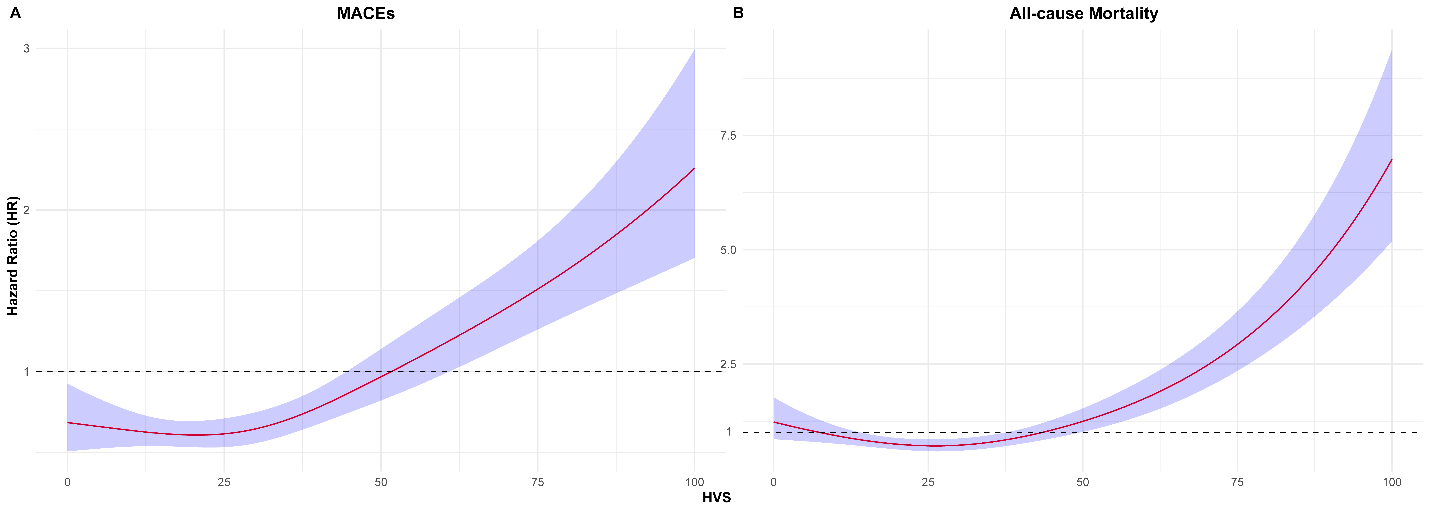


**S3 Fig.** Restricted cubic splines (RCS) revealed the non-linear relationship between HVS and outcomes. The solid red line is the estimated HR and the purple interval is the 95% confidence interval.
