# Supplementary material for: Association between estimated glucose disposal rate and major adverse cardiovascular events in patients with type 2 diabetes
Source: PLoS One. 2025 Jul 17;20(7):e0328252. doi: 10.1371/journal.pone.0328252 (PMC12270132; doi:10.1371/journal.pone.0328252)
Supplement: S4 Fig — Each stratification was adjusted for all factors in model 3 except for the stratification factor itself. CI, confidence interval. (DOCX) [file pone.0328252.s004.docx]

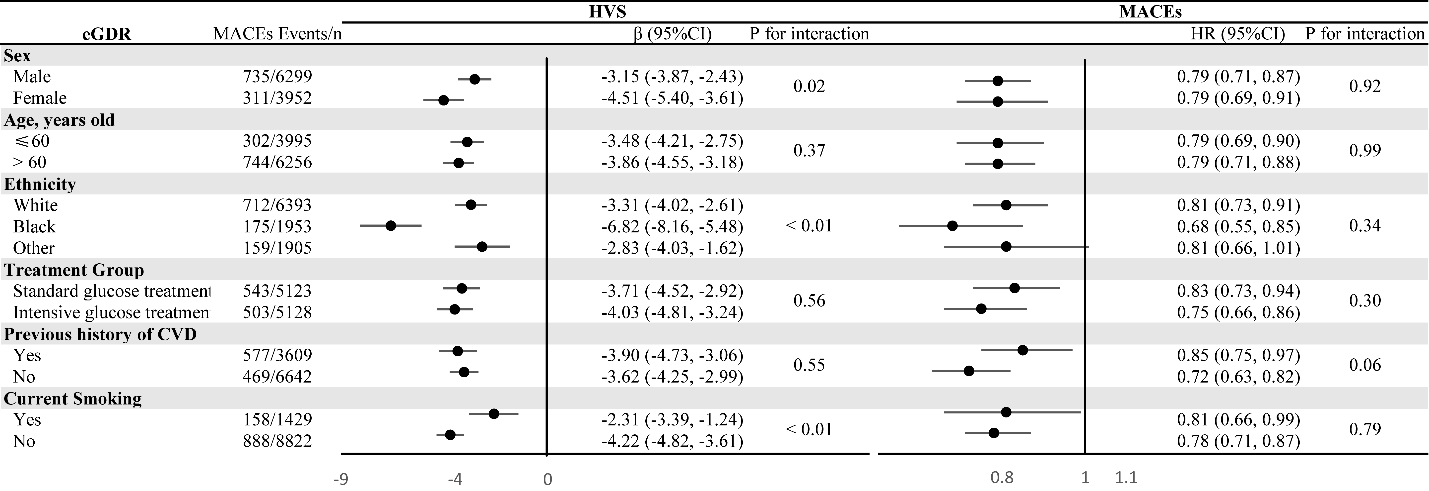


**S4 Fig.** β or hazard ratios per 1 standard deviation–increase in eGDR for HVS and major adverse cardiovascular events. Each stratification was adjusted for all factors in model 3 except for the stratification factor itself. CI, confidence interval.
