# Supplementary material for: Association between estimated glucose disposal rate and major adverse cardiovascular events in patients with type 2 diabetes
Source: PLoS One. 2025 Jul 17;20(7):e0328252. doi: 10.1371/journal.pone.0328252 (PMC12270132; doi:10.1371/journal.pone.0328252)
Supplement: S5 Table — (DOCX) [file pone.0328252.s005.docx]

**S5 Table. Relationship between HVS group and outcomes stratifying participants by time-weighted mean value of HbA1c (≤7% vs >7%) during the trial period.**

| eGDR Tertile | Hazard ratio (95% CI) *P*-Value | | |
| --- | --- | --- | --- |
|  | MACEs | | |
|  | Time-weighted mean value of HbA1c < 7 | Time-weighted mean value of HbA1c >= 7 | P for Interaction |
| T1 | Ref. | Ref. | 0.58 |
| T2 | 1.43 (1.09, 1.89) *P*<0.01 | 1.17 (0.94, 1.46) *P*=0.16 |  |
| T3 | 1.79 (1.28, 2.51) *P*<0.01 | 1.51 (1.16, 1.96) *P*<0.01 |  |
| *P* for trend | <0.01 | <0.01 |  |
|  | All-cause mortality | | |
| T1 | Ref. | Ref. | 0.86 |
| T2 | 1.66 (1.22, 2.26) *P*<0.01 | 1.51 (1.10, 2.07) *P=*0.01 |  |
| T3 | 2.12 (1.46, 3.09) *P*<0.01 | 1.78 (1.23, 2.58) *P*<0.01 |  |
| *P* for trend | <0.01 | <0.01 |  |
