# Supplementary material for: Association between estimated glucose disposal rate and major adverse cardiovascular events in patients with type 2 diabetes
Source: PLoS One. 2025 Jul 17;20(7):e0328252. doi: 10.1371/journal.pone.0328252 (PMC12270132; doi:10.1371/journal.pone.0328252)
Supplement: S6 Table — (DOCX) [file pone.0328252.s006.docx]

**S6 Table. Causal mediation analysis estimates* after including** **time-weighted mean value of HbA1c.**

| eGDR T3 *vs.* T1 | Hazard ratio (95% CI) P-Value | |
| --- | --- | --- |
|  | MACEs | All-cause mortality |
|  | Mediator: HVS | Mediator: HVS |
| Overall |  |  |
| Total effect | 1.37 (1.12, 1.69) *P*<0.01 | 1.49 (1.25, 1.78) *P*<0.01 |
| Natural direct effect | 1.22 (0.98, 1.51) *P*=0.08 | 1.37 (1.14, 1.65) *P*<0.01 |
| Natural indirect effect | 1.13 (1.09, 1.17) *P*<0.01 | 1.09 (1.06, 1.12) *P*<0.01 |
| % mediated | 34.30, *P*=0.03 | 18.40, *P*<0.01 |
| Standard blood glucose management | |  |
| Total effect | 1.42 (1.17, 1.72) *P*<0.01 | 1.47 (1.25, 1.73) *P*<0.01 |
| Natural direct effect | 1.25 (1.02, 1.53) *P*=0.03 | 1.33 (1.12, 1.58) *P*<0.01 |
| Natural indirect effect | 1.14 (1.10, 1.18) *P*<0.01 | 1.10 (1.07, 1.14) *P*<0.01 |
| % mediated | 32.52, *P*=0.01 | 22.14, *P*<0.01 |
| Intensive blood glucose management | |  |
| Total effect | 1.36 (1.09, 1.69) *P*<0.01 | 1.51 (1.25, 1.82) *P*<0.01 |
| Natural direct effect | 1.20 (0.95, 1.51) *P*=0.13 | 1.36 (1.12, 1.66) *P*<0.01 |
| Natural indirect effect | 1.14 (1.09, 1.18) *P*<0.01 | 1.10 (1.07, 1.14) *P*<0.01 |
| % mediated | 37.93, *P*=0.04 | 20.53, *P*<0.01 |

The total effect hazard ratio (HR) represents the overall effect of T3 compared with T1 on the adverse outcomes. It decomposes as follows: (total effect HR) = (natural direct effect HR) × (natural indirect effect HR). CI, confidence interval.

*, Conditioning on overall population median age, body mass index, blood pressure, lipid profile, Estimated Glomerular Filtration Rate, and White, male, smoker, free of comorbidities, time-weighted mean value of HbA1c was set as 7.5% in standard arm and 6.5% in intensive arm.
