# Supplementary material for: Association between estimated glucose disposal rate and major adverse cardiovascular events in patients with type 2 diabetes
Source: PLoS One. 2025 Jul 17;20(7):e0328252. doi: 10.1371/journal.pone.0328252 (PMC12270132; doi:10.1371/journal.pone.0328252)
Supplement: S7 Table — (DOCX) [file pone.0328252.s007.docx]

**S7 Table. Relationship between eGDR tertiles, HVS group and outcomes after adjusting time-weighted mean value of HbA1c.**

| **eGDR Tertile** | Hazard ratio (95% CI) *P*-Value | |  |
| --- | --- | --- | --- |
|  |  |  |  |
|  | MACEs* | All-cause mortality* |  |
| T1 | Ref. | Ref. |  |
| T2 | 1.21 (1.02, 1.44) *P*=0.03 | 1.41 (1.13, 1.75) *P*<0.01 |  |
| T3 | 1.45 (1.18, 1.78) *P*<0.01 | 1.62 (1.24, 2.10) *P*<0.01 |  |
| *P* for trend | <0.01 | <0.01 |  |
| **HVS Group** |  | |  |
| 0-20 | Ref. | Ref. |  |
| 20-40 | 1.19 (0.98, 1.44) *P*=0.08 | 1.17 (0.92, 1.49) *P=*0.21 |  |
| 40-60 | 2.00 (1.65, 2.44) *P*<0.01 | 1.82 (1.42, 2.34) *P*<0.01 |  |
| 60-80 | 1.98 (1.55, 2.52) *P*<0.01 | 2.95 (2.22, 3.93) *P*<0.01 |  |
| 80-100 | 3.98 (3.02, 5.23) *P*<0.01 | 6.42 (4.71, 8.75) *P*<0.01 |  |
| *P* for trend | <0.01 | <0.01 |  |

*, we used the model 3 plus time-weighted mean value of HbA1c.

Model 3 was the full-adjusted model, adjusted for age, sex, ethnicity, CVD history, treatment arm, body mass index (BMI), blood pressure, hyperlipidemia, estimated glomerular filtration rate, comorbidity (heart failure, depression, albuminuria), and smoking status.

CI, confidence interval.
