# Supplementary material for: Association between estimated glucose disposal rate and major adverse cardiovascular events in patients with type 2 diabetes
Source: PLoS One. 2025 Jul 17;20(7):e0328252. doi: 10.1371/journal.pone.0328252 (PMC12270132; doi:10.1371/journal.pone.0328252)
Supplement: S9 Table — (DOCX) [file pone.0328252.s009.docx]

**S9 Table. Causal mediation analysis estimates*, by conditioning on Black people.**

| eGDR T3 *vs.* T1 | Hazard ratio (95% CI) P-Value | |
| --- | --- | --- |
|  | MACEs | All-cause mortality |
|  | Mediator: HVS | Mediator: HVS |
| Overall |  |  |
| Total effect | 1.39 (1.14, 1.70) *P*<0.01 | 1.51 (1.26, 1.80) *P*<0.01 |
| Natural direct effect | 1.21 (0.98, 1.50) *P*=0.08 | 1.36 (1.13, 1.64) *P*<0.01 |
| Natural indirect effect | 1.14 (1.10, 1.19) *P*<0.01 | 1.11 (1.08, 1.15) *P*<0.01 |
| % mediated | 37.15, *P*=0.02 | 21.80, *P*<0.01 |
| Standard blood glucose management | |  |
| Total effect | 1.42 (1.18, 1.72) *P*<0.01 | 1.47 (1.24, 1.73) *P*<0.01 |
| Natural direct effect | 1.24 (1.02, 1.51) *P*=0.03 | 1.31 (1.10, 1.55) *P*<0.01 |
| Natural indirect effect | 1.15 (1.10, 1.19) *P*<0.01 | 1.12 (1.09, 1.16) *P*<0.01 |
| % mediated | 34.88, *P*<0.01 | 26.01, *P*<0.01 |
| Intensive blood glucose management | |  |
| Total effect | 1.36 (1.10, 1.69) *P*<0.01 | 1.52 (1.26, 1.85) *P*<0.01 |
| Natural direct effect | 1.19 (0.94, 1.49) *P*=0.15 | 1.36 (1.11, 1.66) *P*<0.01 |
| Natural indirect effect | 1.15 (1.10, 1.19) *P*<0.01 | 1.12 (1.09, 1.16) *P*<0.01 |
| % mediated | 40.78, *P*=0.04 | 23.16, *P*<0.01 |

The total effect hazard ratio (HR) represents the overall effect of T3 compared with T1 on the adverse outcomes. It decomposes as follows: (total effect HR) = (natural direct effect HR) × (natural indirect effect HR). CI, confidence interval.

*, Conditioning on overall population median age, body mass index, blood pressure, lipid profile, Estimated Glomerular Filtration Rate, and Black, male, non-smoker, free of comorbidities.
