# Supplementary material for: Association between estimated glucose disposal rate and major adverse cardiovascular events in patients with type 2 diabetes
Source: PLoS One. 2025 Jul 17;20(7):e0328252. doi: 10.1371/journal.pone.0328252 (PMC12270132; doi:10.1371/journal.pone.0328252)
Supplement: S10 Table — (DOCX) [file pone.0328252.s010.docx]

**S10 Table. Causal mediation analysis estimates*, by conditioning on ethnicity (non-White and non-Black).**

| eGDR T3 *vs.* T1 | Hazard ratio (95% CI) P-Value | |
| --- | --- | --- |
|  | MACEs | All-cause mortality |
|  | Mediator: HVS | Mediator: HVS |
| Overall |  |  |
| Total effect | 1.41 (1.16, 1.71) *P*<0.01 | 1.49 (1.26, 1.77) *P*<0.01 |
| Natural direct effect | 1.23 (1.00, 1.51) *P*=0.05 | 1.34 (1.12, 1.61) *P*<0.01 |
| Natural indirect effect | 1.15 (1.10, 1.19) *P*<0.01 | 1.11 (1.08, 1.15) *P*<0.01 |
| % mediated | 35.65, *P*=0.01 | 22.61, *P*<0.01 |
| Standard blood glucose management | |  |
| Total effect | 1.44 (1.20, 1.73) *P*<0.01 | 1.45 (1.24, 1.71) *P*<0.01 |
| Natural direct effect | 1.25 (1.04, 1.52) *P*=0.02 | 1.30 (1.10, 1.53) *P*<0.01 |
| Natural indirect effect | 1.15 (1.10, 1.19) *P*<0.01 | 1.12 (1.09, 1.16) *P*<0.01 |
| % mediated | 33.61, *P*<0.01 | 26.98, *P*<0.01 |
| Intensive blood glucose management | |  |
| Total effect | 1.38 (1.12, 1.69) *P*<0.01 | 1.51 (1.26, 1.81) *P*<0.01 |
| Natural direct effect | 1.20 (0.96, 1.50) *P*=0.10 | 1.35 (1.11, 1.63) *P*<0.01 |
| Natural indirect effect | 1.15 (1.10, 1.19) *P*<0.01 | 1.12 (1.09, 1.16) *P*<0.01 |
| % mediated | 39.09, *P*=0.03 | 24.01, *P*<0.01 |

The total effect hazard ratio (HR) represents the overall effect of T3 compared with T1 on the adverse outcomes. It decomposes as follows: (total effect HR) = (natural direct effect HR) × (natural indirect effect HR). CI, confidence interval.

*, Conditioning on overall population median age, body mass index, blood pressure, lipid profile, Estimated Glomerular Filtration Rate, and other ethnicity, male, non-smoker, free of comorbidities.
