# Supplementary material for: Association between estimated glucose disposal rate and major adverse cardiovascular events in patients with type 2 diabetes
Source: PLoS One. 2025 Jul 17;20(7):e0328252. doi: 10.1371/journal.pone.0328252 (PMC12270132; doi:10.1371/journal.pone.0328252)
Supplement: S11 Table — (DOCX) [file pone.0328252.s011.docx]

**S11 Table. Causal mediation analysis estimates*, by conditioning on smoker.**

| eGDR T3 *vs.* T1 | Hazard ratio (95% CI) P-Value | |
| --- | --- | --- |
|  | MACEs | All-cause mortality |
|  | Mediator: HVS | Mediator: HVS |
| Overall |  |  |
| Total effect | 1.39 (1.14, 1.70) *P*<0.01 | 1.51 ( 1.26, 1.80) *P*<0.01 |
| Natural direct effect | 1.22 (0.99, 1.50) *P*=0.07 | 1.36 (1.13, 1.63) *P*<0.01 |
| Natural indirect effect | 1.15 (1.10, 1.19) *P*<0.01 | 1.11 (1.08, 1.15) *P*<0.01 |
| % mediated | 36.85, *P*=0.02 | 22.05, *P*<0.01 |
| Standard blood glucose management | |  |
| Total effect | 1.43 (1.18, 1.72) *P*<0.01 | 1.47 (1.24, 1.73) *P*<0.01 |
| Natural direct effect | 1.24 (1.02, 1.51) *P*=0.03 | 1.31 (1.10, 1.55) *P*<0.01 |
| Natural indirect effect | 1.15 (1.10, 1.19) *P*<0.01 | 1.11 (1.08, 1.15) *P*<0.01 |
| % mediated | 34.60, *P*<0.01 | 26.34, *P*<0.01 |
| Intensive blood glucose management | |  |
| Total effect | 1.37 (1.10, 1.69) *P*<0.01 | 1.52 (1.26, 1.84) *P*<0.01 |
| Natural direct effect | 1.19 (0.95, 1.49) *P*=0.13 | 1.36 (1.11, 1.65) *P*<0.01 |
| Natural indirect effect | 1.15 (1.10, 1.19) *P*<0.01 | 1.11 (1.08, 1.15) *P*<0.01 |
| % mediated | 40.37, *P*=0.03 | 23.48, *P*<0.01 |

The total effect hazard ratio (HR) represents the overall effect of T3 compared with T1 on the adverse outcomes. It decomposes as follows: (total effect HR) = (natural direct effect HR) × (natural indirect effect HR). CI, confidence interval.

*, Conditioning on overall population median age, body mass index, blood pressure, lipid profile, Estimated Glomerular Filtration Rate, and White, male, smoker, free of comorbidities.
